# A Case of Systemic Lupus Erythematosus Initially Presenting With Generalized Lymphadenopathy: A Diagnostic Challenge

**DOI:** 10.7759/cureus.106588

**Published:** 2026-04-07

**Authors:** Eugenio Vázquez-Meraz, José Arellano-Galindo, Alberto Díaz-Romero, Jorge A Pérez-Castro, Carolina Duarte-Salazar

**Affiliations:** 1 Hematology, Hospital General de México, Mexico City, MEX; 2 Virology, Hospital Infantil De México Federico Gómez, Mexico City, MEX; 3 Medicine, Centro Interidisciplinario de Ciencias de la Salud Unidad Milpa Alta del Instituto Politécnico Nacional, Mexico City, MEX; 4 Infectology, Hospital Angeles Metropolitano, Mexico City, MEX; 5 Pathology, Hospital Angeles Metropolitano, Mexico City, MEX; 6 Rheumatology, Instituto Nacional de Rehabilitación Luis Guillermo Ibarra Ibarra (LGII), Mexico City, MEX

**Keywords:** arthritis, autoimmune disease, differential diagnosis: autoimmune disease, generalized lymphadenopathy, inflammation, systemic lupus erythematosus

## Abstract

Lymphadenopathy (LAD) can emerge as an initial manifestation of systemic lupus erythematosus (SLE); however, establishing an accurate differential diagnosis remains challenging due to the wide spectrum of infectious, malignant, and autoimmune conditions associated with this presentation. We report the case of a 47-year-old man admitted with a two-month history of persistent fever (approximately 38 °C), night sweats, chills, and diffuse generalized lymphadenopathy. Physical examination revealed enlarged, mobile, non-tender, soft cervical, axillary, and inguinal lymph nodes measuring approximately 2-3 cm in diameter, along with hepatomegaly and splenomegaly. An extensive diagnostic workup was initially undertaken to exclude malignant and infectious causes. Two weeks after admission, the patient developed inflammatory arthritis, hemolytic anemia, severe thrombocytopenia, leukopenia, and lymphopenia. Given the evolving clinical findings, SLE was suspected, and comprehensive lupus-specific serological testing confirmed the diagnosis. The patient fulfilled the 2019 American College of Rheumatology (ACR)/European League Against Rheumatism (EULAR) classification criteria for SLE based on immunological abnormalities and multisystem involvement. Treatment with high-dose corticosteroids, intravenous immunoglobulin, and immunosuppressive therapy led to significant clinical improvement. This case underscores that extensive generalized LAD may represent an initial presentation of SLE and should be included in the differential diagnosis of patients presenting with LAD and constitutional symptoms.

## Introduction

Lymphadenopathy (LAD) may occur in the context of immunological diseases, particularly rheumatoid arthritis, systemic lupus erythematosus (SLE), and Sjögren’s syndrome. In addition to these autoimmune disorders, other etiologies of LAD, including malignant diseases and infections, must also be considered [1,2]. In patients with SLE, LAD is a common clinical manifestation, with a reported prevalence ranging from 33% to 69%. It is more frequently generalized; however, localized LAD has been described in up to 50% of cases [3]. Typically, lymph nodes affected in SLE are soft, non-tender, and measure less than 1 cm in diameter [4]. Histopathological findings commonly reveal reactive follicular hyperplasia, hematoxylin bodies, and varying degrees of coagulative necrosis [5]. Notably, LAD is often associated with active disease in SLE [6]. This case report describes a patient in whom generalized LAD constituted the initial manifestation of SLE, highlighting the diagnostic challenges involved in establishing a definitive diagnosis.

## Case presentation

**Figure 1:**
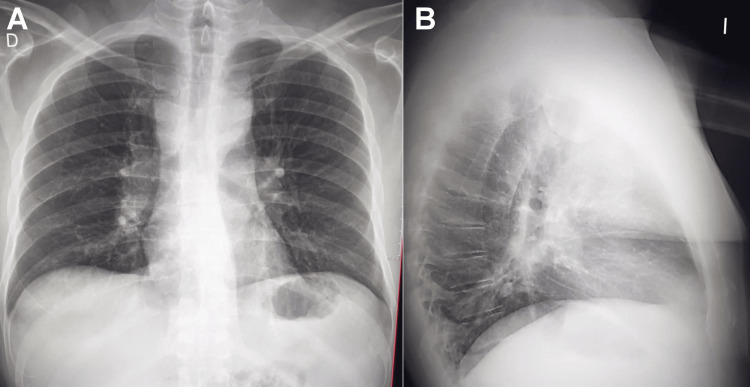
Posteroanterior and lateral chest radiographs demonstrating (A) mediastinal widening with prominence of the aortopulmonary window; (B) findings consistent with hilar lymphadenopathy.

A 47-year-old man was admitted with a two-month history of persistent fever (approximately 38 °C), night sweats, chills, and generalized LAD. Physical examination revealed enlarged, mobile, non-tender, soft lymph nodes in the cervical, axillary, and inguinal regions, measuring approximately 2-3 cm in diameter. Hepatomegaly and splenomegaly were also noted. To evaluate systemic inflammation, laboratory studies were done which demonstrated significant inflammatory findings, including a high-sensitivity C‑reactive protein level of 8.5 mg/L (reference values commonly used in rheumatology: < 1.0 mg/L, no inflammation; 1.0-3.0 mg/L, mild or subclinical inflammation; 3.0-10 mg/L, low to moderate active inflammation; > 10 mg/L, significant active inflammation) and an erythrocyte sedimentation rate of 63 mm/h. Given concern for an underlying malignancy, particularly lymphoma, a hematology consultation was obtained. Chest radiography revealed bilateral hilar LAD (Figure 1).

**Figure 2:**
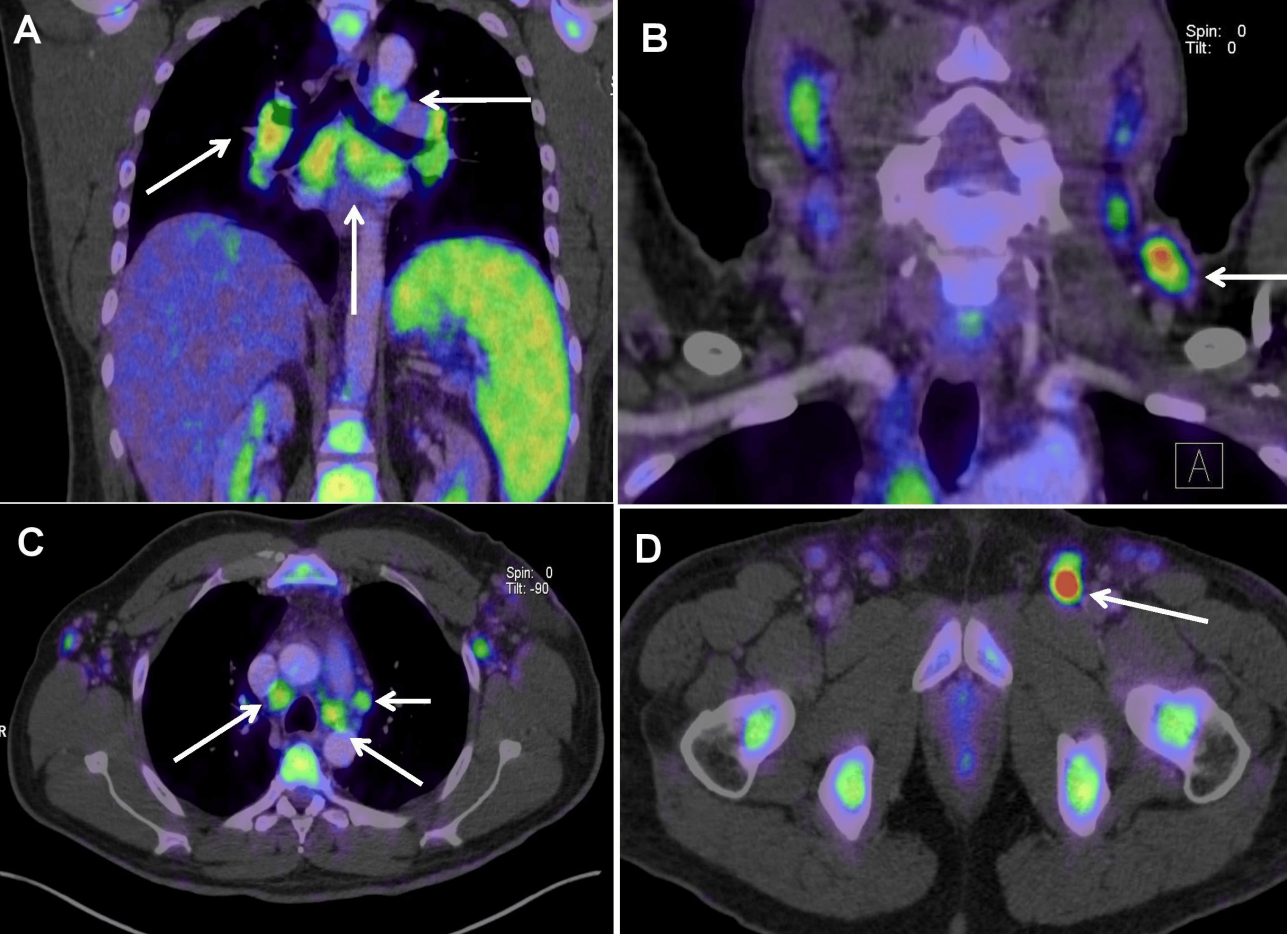
PET/CT showing multifocal lymphadenopathy (white arrows): (A) coronal plane with mediastinal and hilar involvement and splenomegaly; (B–D) axial planes showing cervical, paratracheal, paraaortic, axillary, and inguinal lymphadenopathy.

Positron emission tomography/computed tomography (PET/CT) confirmed widespread LAD involving the paratracheal, cervical, mediastinal, hilar, axillary, and inguinal regions (Figure 2).

**Figure 3:**
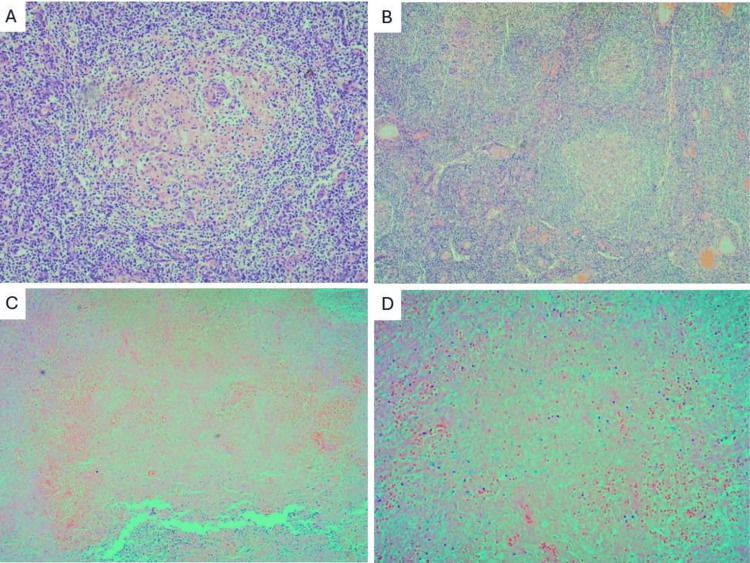
Histopathological analysis of cervical and axillary lymph nodes revealed several notable findings A) Lymphoid follicles displaying a germinal proliferative pattern (10X magnification); B) Follicular lymphoid hyperplasia characterized by a proliferative pattern (4X magnification); C) Evidence of a necrotizing pattern (4X magnification); and D) Coagulative necrosis (10X magnification). Collectively, these histological features are indicative of systemic lupus erythematosus.

Excisional biopsies of cervical and inguinal lymph nodes revealed no evidence of lymphoma. Histopathological examination demonstrated lymphoid follicles with proliferative germinal centers, necrotizing follicular lymphoid hyperplasia, and areas of coagulative necrosis, findings suggestive of SLE (Figure 3). Kikuchi-Fujimoto disease (KFD) was considered in the differential diagnosis due to its well‑recognized clinical and histopathological overlap with SLE. However, KFD was ruled out based on the histopathological features observed, as the biopsy did not demonstrate any of the three classical KFD patterns, proliferative, necrotizing, or xanthomatous, thereby excluding this entity from the differential diagnosis. Although KFD is frequently associated with fever, cervical LAD, arthralgias, and leukopenia, all of which may also be present in SLE, the overall clinicopathological correlation was more consistent with SLE [7]. Regarding laboratory findings, the patient fulfilled the 2019 American College of Rheumatology (ACR)/European League Against Rheumatism (EULAR) classification criteria for SLE [8]. The mandatory entry criterion of a positive antinuclear antibody (ANA) titer ≥ 1:80 was met. In addition, additive criteria from both clinical and immunological domains yielded a total score exceeding 10 points. These included constitutional involvement manifested by fever (2 points); hematologic involvement, including leukopenia, thrombocytopenia, and autoimmune hemolytic anemia (4 points); musculoskeletal involvement with synovitis (6 points); and immunological criteria, including antiphospholipid antibodies (anticardiolipin (aCL) antibodies, anti‑β2‑glycoprotein I antibodies, or lupus anticoagulant) (2 points), SLE‑specific antibodies such as anti‑double‑stranded DNA (anti‑dsDNA) antibodies (6 points), and hypocomplementemia with decreased C3 and C4 levels (4 points) [9].

**Figure 4:**
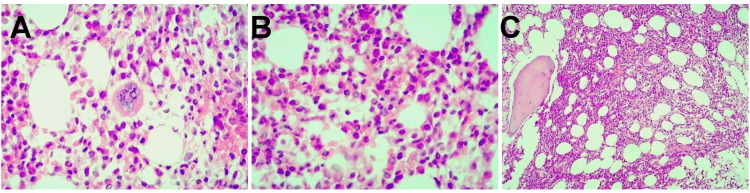
Normocellular bone marrow biopsy stained with hematoxylin and eosin. (A) Bone marrow cellularity of approximately 70% (10×); (B and C) Preserved megakaryocytic lineage, with granulocytic and erythroid lineages showing no dysplastic changes (40×).

Bone marrow biopsy revealed normocellular marrow without evidence of malignancy or hematolymphoid infiltration (Figure 4).

Infectious etiologies were comprehensively excluded. Blood and urine cultures were sterile. Evaluation for *Mycobacterium tuberculosis*, including purified protein derivative, QuantiFERON-TB Gold assay, and polymerase chain reaction analysis of lymph node tissue, was negative. Serological testing for Epstein-Barr virus, cytomegalovirus, herpes simplex virus types 1 and 2, *Toxoplasma gondii*, measles virus, hepatitis B and C viruses, HIV, and SARS-CoV-2 was also negative.

Two weeks into hospitalization, the patient’s clinical condition deteriorated with the development of inflammatory arthritis, hemolytic anemia, severe thrombocytopenia, leukopenia, and lymphopenia, further supporting the suspicion of SLE (Table 1). Laboratory evaluation did not demonstrate abnormalities typically observed in macrophage activation syndrome, such as elevated serum levels of aspartate aminotransferase, lactate dehydrogenase, hypertriglyceridemia, or markedly increased serum ferritin levels, which represent a key diagnostic finding. Additionally, bone marrow examination did not reveal evidence of hemophagocytosis.

**Table 1:** Hematological parameters observed during the second and third weeks of hospitalization

Laboratory Parameter	baseline	Two Weeks	Three Weeks	Reference Range
Hemoglobin (g/dL)	13.7	9	10.4	14–18
Indirect Bilirubin (mg/dL)	0.76	1.47	0.74	0.0–1.0
Lactate dehydrogenase (U/L)	127	335	134	125–240
Reticulocytes (%)	0.5	8.43	0.68	0.5–1.5
Leukocytes (×10³/µL)	4.5	2.57	4.37	3.80–11.20
Lymphocytes (×10³/µL)	1480	0.49	1.11	0.80–4.50
Platelets (×10³/µL)	150	9	195	130–400

Immunological testing revealed a positive ANA assay with a nuclear pattern on HEp-2 cells, positive anti-double-stranded DNA (anti-dsDNA) antibodies detected by enzyme-linked immunosorbent assay, and elevated aCL antibodies of both IgM and IgG isotypes. The direct Coombs test was positive, and complement levels (C3 and C4) were decreased, findings that further supported the diagnosis of SLE (Table 2).

**Table 2:** Immunological test results (at the initial and six-month follow-up). Anti-dsDNA antibodies were considered positive at a titer dilution ≥ 1:320. *Anticardiolipin IgM antibodies were measured in IgM phospholipid units (MPL). **Not determined. ***Anticardiolipin IgG antibodies (aCL IgG). ****IgM antibodies directed against the phosphatidylserine–prothrombin (PS/PT) complex.

Test	Baseline	6 Months follow-up	Reference
ANA (IF, nuclear pattern)	2:40	Negative	Negative
Anti-dsDNA	Positive	Negative	Negative
aCL IgM (MPL)*	75.4	ND**	0.0–19.9
aCL IgG (GPL)***	28.2	ND**	0.0–19.9
PS/PT IgM (U)****	33.7	ND**	<30.0
Direct Coombs	3:08	1:32	Negative
Complement C3 (mg/dL)	57	83	82.0–185.0
Complement C4 (mg/dL)	7.2	29.9	15.0–53.0

Other autoantibodies, including anti-Sm, anti-Ro, anti-La, anti-β2-glycoprotein I (IgA, IgG, and IgM), lupus anticoagulant (dilute Russell viper venom time (dRVVT) method), and antiplatelet antibodies, were negative.

The patient received intravenous methylprednisolone at a dose of 1 g daily for three consecutive days, followed by oral prednisone at 1 mg/kg/day. Due to persistent severe thrombocytopenia with inadequate response to corticosteroid therapy, intravenous immunoglobulin was administered at a dose of 400 mg/kg/day for five consecutive days. Subsequently, treatment with rituximab was initiated at a dose of 750 mg intravenously once weekly for four consecutive weeks. Following this therapeutic approach, marked clinical, hematological, and immunological improvement was observed. The patient remained in clinical remission and was maintained on supportive therapy with hydroxychloroquine (200 mg/day), mycophenolate mofetil (2 g/day), and low‑dose prednisone (10 mg/day).

## Discussion

LAD is a common manifestation in rheumatologic diseases, including SLE [4,5]. However, its evaluation remains challenging due to the broad differential diagnosis, which includes lymphoproliferative disorders, infections, and other autoimmune diseases. Consequently, the initial diagnostic workup of patients presenting with LAD should prioritize the exclusion of malignancy and infectious etiologies [1,4].

In patients with SLE, LAD has been reported in up to 69% of cases, most frequently presenting as generalized LAD [3]. Although often considered a benign clinical feature, its presence may correlate with increased disease activity and, in certain cases, require more aggressive immunosuppressive therapy. Histopathological findings in SLE‑associated LAD typically include reactive follicular hyperplasia and other reactive changes; however, atypical or proliferative patterns have also been described [6,7].

KFD represents a particularly important entity in the differential diagnosis, owing to its clinical and histopathological overlap with SLE. KFD is commonly associated with fever, cervical lymphadenopathy, arthralgias, and leukopenia, features that may also be observed in SLE. Regarding laboratory findings, Nishimura et al. evaluated 112 patients with KFD and reported that eight of 46 patients tested (17%) had a positive ANA, defined as a titer > 1:40; however, the absolute ANA titers were only mildly elevated, with a mean value of 1:50 ± 17.3. Additionally, anti‑Sm, anti‑dsDNA, and anticardiolipin antibodies were each detected in only one patient. Importantly, histological features typical of SLE were not observed, and no significant differences were identified between autoantibody‑positive and autoantibody‑negative KFD patients. None of these individuals fulfilled the classification criteria for autoimmune diseases [8,9].

The definitive diagnosis of KFD relies on histopathological examination, with three recognized patterns: proliferative, necrotizing, and xanthomatous. The proliferative pattern is characterized by an admixture of histiocytes, plasmacytoid dendritic cells, and lymphocytes within a background of karyorrhectic nuclear debris and eosinophilic material. The necrotizing pattern exhibits variable degrees of coagulative necrosis within cellular aggregates, whereas the xanthomatous pattern is defined by the predominance of foamy histiocytes, regardless of the presence or absence of necrosis. The necrotizing pattern is further distinguished by abundant apoptotic debris, cells at various stages of apoptosis, and numerous perinuclear vacuoles [8,9].

In contrast, the patient described in this report fulfilled the 2019 EULAR/ACR classification criteria for SLE [8]. The mandatory entry criterion of ANA positivity at a titer ≥ 1:80 was met. Furthermore, additive criteria from both clinical and immunological domains yielded a cumulative score exceeding 10 points. These included constitutional involvement manifested by fever (2 points); hematologic involvement with leukopenia, thrombocytopenia, and autoimmune hemolytic anemia (4 points); musculoskeletal involvement with synovitis (6 points); and immunological criteria, including antiphospholipid antibodies (anticardiolipin antibodies, anti‑β2‑glycoprotein I antibodies, or lupus anticoagulant) (2 points), SLE‑specific antibodies such as anti‑double‑stranded DNA antibodies (6 points), and hypocomplementemia with decreased C3 and C4 levels (4 points). This case highlights generalized lymphadenopathy as the initial manifestation of SLE, a rare but well-recognized clinical scenario [10-12]. The patient fulfilled the 2019 EULAR/ACR classification criteria for SLE, accumulating a total of 18 points across clinical, hematological, and immunological domains (fever: 2 points; arthritis: 6 points; leukopenia, hemolytic anemia, and thrombocytopenia: 4 points; low C3/C4 levels and anticardiolipin antibodies: 6 points) [8]. Notably, despite its clinical relevance, lymphadenopathy is not included in the current classification criteria. Mediastinal and hilar LAD as initial manifestations of SLE are particularly uncommon and are more frequently associated with infectious processes or lymphoma. This underscores the importance of lymph node biopsy in atypical presentations to avoid misdiagnosis and to guide appropriate therapeutic decisions [10].

Previous reports, including a pediatric case of ANA-negative SLE, have demonstrated that patients presenting with generalized LAD may lack typical serological markers, thereby further complicating the differential diagnosis between SLE and lymphoproliferative disorders [13]. These observations indicate that clinical and immunological manifestations may vary considerably among patients and, under certain circumstances, can result in diagnostic uncertainty, underscoring the need for heightened clinical vigilance. In this context, early and accurate diagnosis of SLE is essential to reduce the risk of major organ involvement, prevent irreversible damage accrual, and improve overall outcomes, as delayed or incorrect diagnoses have been associated with severe complications such as lupus nephritis or nephrotic syndrome and poorer prognoses [13].

There have been no systematic studies of lymphatic function in SLE to date. The lymphatic system plays an important role in autoimmune diseases, considering lymph nodes as possible gatekeepers of autoimmunity [13]. Because lymphatic vessels are essential for clearing tissue waste, transporting antigens, and mediating immune response, it may not be surprising that the lymphatics are involved in the pathophysiology of autoimmune diseases. The lymphatic system directly participates in immune activation and modulation by facilitating the transport of antigens into the lymph node and subsequently to the site of inflammation [14]. A recent study identified dysfunctional lymphatic flow as one of the factors that contribute to the pathophysiology of SLE [14,15].

## Conclusions

Generalized LAD is an uncommon but clinically significant initial manifestation of SLE. This case highlights the importance of considering SLE in the differential diagnosis of patients presenting with diffuse LAD and constitutional symptoms, particularly when infectious and malignant causes have been excluded, as previously described. Early recognition and appropriate immunological evaluation are essential to avoid diagnostic delays and to facilitate the timely initiation of immunosuppressive therapy, which may lead to favorable clinical outcomes.
